# Three New Calcium Formate Reference Materials for *δ* ^13^C Measurements and a Redetermination of the *R*(^13^C/^12^C) Ratio for VPDB Based on Proton Nuclear Magnetic Resonance Measurements

**DOI:** 10.1002/rcm.70132

**Published:** 2026-07-01

**Authors:** David W. Hoffman, Cornelia Rasmussen, Arndt Schimmelmann, Lauren T. Reid, Haiping Qi, Tyler B. Coplen

**Affiliations:** ^1^ Department of Molecular Biosciences, College of Natural Science University of Texas at Austin Austin Texas USA; ^2^ University of Texas Institute for Geophysics, Jackson School of Geosciences University of Texas at Austin Austin Texas USA; ^3^ Department of Earth and Atmospheric Sciences Indiana University Bloomington Indiana USA; ^4^ U.S. Geological Survey Reston Virginia USA

## Abstract

**Rationale:**

Isotope ratio mass spectrometry (IRMS) and proton nuclear magnetic resonance (^1^H NMR) spectroscopy are independent techniques for determining the isotope ratio *R*(^13^C/^12^C) in organic compounds. However, the lack of suitable reference materials has limited intercalibration of results from these methods.

**Methods:**

Three high‐purity calcium formate isotopic reference materials were developed, each ideal for accurate isotope ratio measurements by both ^1^H NMR and isotope‐ratio mass spectrometry (IRMS). For each material, *R*(^13^C/^12^C) was determined by ^1^H NMR, and the relative isotope abundance (*δ* ^13^C_VPDB‐LSVEC_) by IRMS. The combined IRMS and NMR results were used to determine the *R*(^13^C/^12^C) value of Vienna Peedee belemnite (VPDB).

**Results:**

The combined datasets yield a value for *R*(^13^C/^12^C) of 0.0111050 ± 0.0000047 (*k* = 2) for VPDB, which is 6.7‰ lower than the commonly cited value of 0.011180, but similar to other recent determinations. These three calcium formate reference materials exhibit high accuracy across both analytical methods and provide robust tools for instrument calibration, method validation, and inter‐laboratory comparison in carbon stable isotope ratio analysis.

**Conclusions:**

An accurate *R*(^13^C/^12^C) value for VPDB strengthens the link between relative (*δ* ^13^C_VPDB‐LSVEC_) values from IRMS and *R*(^13^C/^12^C) isotope ratios from NMR and other techniques. These three calcium formate isotopic reference materials (USGS106, USGS107, and USGS108) are available from the US Geological Survey and provide reference materials for calibration, method validation, and inter‐laboratory comparison in carbon isotope ratio analysis.

## 1 Introduction

Measurements of the relative abundance of ^13^C and ^12^C (*δ* ^13^C) have an essential role in numerous scientific disciplines [1]. Analyses of *δ* ^13^C values have provided critical insights into Earth's history [2, 3, 4, 5], the earliest evidence of life [6, 7], and paleoclimate and palaeoceanographic changes [8, 9, 10]. Beyond the geosciences, isotope ratio measurements have proven valuable for detecting counterfeit products [11], identifying illicit drug use in sports [12], and for understanding the origin of organics on Mars [13]. The quantity *δ* ^13^C is most commonly determined using isotope ratio mass spectrometry (IRMS), but other techniques are also employed, including secondary ion mass spectrometry (SIMS) [7], proton nuclear magnetic resonance (^1^H NMR) [14], fluorine nuclear magnetic resonance (^19^F NMR) [15, 16], phosphorus nuclear magnetic resonance (^31^P NMR) [17], Fourier transform infrared spectroscopy (FTIR) [18, 19], tunable diode laser absorption spectroscopy (TDLAS) [20], and cavity ring‐down spectroscopy (CRDS) [21].

In 1987 [22], the *δ* ^13^C_VPDB_ isotope‐delta scale was adopted by assigning a consensus permanently fixed *δ* ^13^C_VPDB_ value of +1.95‰ to NBS 19 limestone [23]. NBS 19 is a scale‐defining isotopic reference material, and such reference materials are termed “primary isotopic reference materials” in Brand et al. [24]. To improve comparability among laboratories, a second scale‐defining isotopic reference material, LSVEC lithium carbonate, was introduced and assigned the permanently fixed consensus value of −46.6‰ in 2006 [25, 26]. This isotope‐delta scale is commonly identified as the *δ* ^13^C_VPDB‐LSVEC_ scale. In 2015, LSVEC was found to be unsuitable as a carbon isotopic reference material because its *δ* ^13^C value increased (became less negative) as it absorbed CO_2_ from air [27]. Nevertheless, the *δ* ^13^C_VPDB‐LSVEC_ scale maintains abundant popularity and is the isotope scale of choice for reporting *δ* ^13^C values measured using elemental analyzers (EAs) and many other methods [28]. Laboratories regularly normalize (calibrate) their measurements to the *δ* ^13^C_VPDB‐LSVEC_ scale by using non‐scale‐defining isotopic reference materials (termed secondary isotopic reference materials in Brand et al. [24]). Using the high‐accuracy *δ* ^13^C_VPDB_ measurements of USGS44 of −42.085‰ ± 0.008‰ and of NBS 19 of +1.95‰ ± 0.003‰ in Table 9 of Qi et al. [29], *δ* ^13^C_VPDB_ and *δ* ^13^C_VPDB‐LSVEC_ values expressed on the *δ* ^13^C_VPDB_ and *δ* ^13^C_VPDB‐LSVEC_ scales are related by the relations:

(1) $$\delta^{13}C_{\text{VPDB}}=0.99717\times \delta^{13}C_{\text{VPDB}-\text{LSVEC}}+0.006‰$$

and

(2) $$\delta^{13}C_{\text{VPDB}-\text{LSVEC}}=1.00283\times \delta^{13}C_{\text{VPDB}}–0.006‰$$

We do not use the *δ* ^13^C_VPDB_ values published for IAEA‐610, IAEA‐611, and IAEA‐612 to relate the *δ* ^13^C_VPDB_ and *δ* ^13^C_VPDB‐LSVEC_ scales because their *δ* ^13^C_VPDB_ values [30] are not in agreement with that of USGS44 [28, 29]. Note that the value of −42.085‰ ± 0.008‰ is within the standard uncertainty of −42.073‰ ± 0.015‰ preferred by Moossen et al. [31].

In contrast to the IRMS differential method, other analytical methods such as (^1^H, ^19^F, ^31^P) NMR and FTIR measure the number ratio or amount ratio of ^13^C and ^12^C, *R*(^13^C/^12^C), by comparing signals associated with the ^13^C versus ^12^C isotopologues within a sample. The *R*(^13^C/^12^C) of VPDB, *R*(^13^C/^12^C)_VPDB_, and the quantity *δ* ^13^C_VPDB_ are related by the relation:
(3)

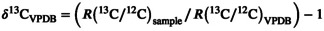

where *R*(^13^C/^12^C)_sample_ is the *R*(^13^C/^12^C) value of a sample and *R*(^13^C/^12^C)_VPDB_ is the *R*(^13^C/^12^C) value of the virtual VPDB reference. Thus, the comparison of results obtained by different analytical methods requires an accurate knowledge of the *R*(^13^C/^12^C)_VPDB_ value.

Both IRMS and ^1^H NMR are established techniques for the determination of carbon stable isotopic compositions [14, 17, 32, 33]. However, the absence of suitable reference materials has limited the application of *R*(^13^C/^12^C) measurements by ^1^H NMR and has hindered intercalibration with IRMS. In addition, the uncertainty in the *R*(^13^C/^12^C) value of VPDB further limits direct comparisons of *R*(^13^C/^12^C) measurements obtained by different techniques. An appropriate reference material must exhibit isotopic homogeneity, chemical stability, and high purity. These properties are critical given that ^1^H NMR provides position‐specific *R*(^13^C/^12^C) values at defined molecular sites, whereas IRMS yields bulk *δ* ^13^C values averaged across all carbon atoms in a sample, including impurities. In addition, an effective reference material should be soluble in a standard NMR solvent, yet remain solid and chemically stable for IRMS analysis, thereby minimizing the risk of isotopic fractionation during storage or measurement. Ideally, the compound should contain a single carbon atom directly bonded to a proton, permitting direct comparison between the position‐specific values obtained by ^1^H NMR and the bulk *δ* ^13^C values determined by IRMS. Calcium formate powder satisfies all these criteria.

Here, we describe the preparation and characterization of three calcium formate isotopic reference materials having a substantial range of carbon isotopic compositions. For each material, we report an *R*(^13^C/^12^C) value determined from ^1^H NMR measurements, its *δ* ^13^C_VPDB‐LSVEC_ value as determined by IRMS, and its *δ* ^13^C_VPDB_ value using Equation (3). Results can aid in comparing future measurements obtained using methods that provide a carbon isotope abundance relative to VPDB, with measurements obtained by ^1^H NMR and other spectroscopic methods that provide *R*(^13^C/^12^C) measurements. These new reference materials are available to other researchers from the US Geological Survey (USGS).

## 2 Materials and Methods

### 2.1 Materials

Three kilograms of BioUltra grade calcium formate Ca(HCOO)_2_ were obtained from Sigma Aldrich, and formic acid (*R* ^13^C/^12^C = 0.99 mol/mol) was obtained from Cambridge Isotope Laboratories (Manchester, New Hampshire). The calcium formate was divided into three fractions. Using the validated method described by Schimmelmann et al. [34] for producing isotopically homogeneous materials, calcium formate was dissolved in ultrapure water. One fraction remained unspiked (USGS106). The other two calcium formate solutions were spiked with small amounts of ^13^C‐enriched calcium formate that had been prepared from the ^13^C‐enriched formic acid via reaction with pure calcium hydroxide to pH 5.02. The isotopic spikes maintained the calcium/formate molar ratio and produced calcium formate solutions that were enriched in ^13^C by approximately 18‰ and 40‰, compared with the approximately −28‰ unspiked solution, resulting in *δ* ^13^C_VPDB‐LSVEC_ values of approximately −10‰ (USGS107) and +14‰ (USGS108). The spiked and unspiked calcium formate solutions were then individually fed through Teflon capillaries into liquid nitrogen to generate small icy droplets that were freeze‐dried to generate isotopically homogeneous fine‐grained powders. The solid calcium formates were analyzed by IRMS to determine their *δ* ^13^C values relative to the VPDB‐LSVEC scale and by ^1^H NMR to determine their *R*(^13^C/^12^C) ratios.

### 2.2 IRMS Analysis

Samples of the three calcium formate isotopic reference materials were analyzed at the Reston Stable Isotope Laboratory of the USGS. The samples were aliquots taken from individual units that had been subsampled from the bulk material. Samples were weighed to introduce a consistent carbon mass (81.6 μg) into the mass spectrometer. Powdered samples were converted to CO_2_ in an EA (Costech, Valencia, CA, United States) that was connected to a Thermo Fisher Scientific ConFlo IV interface, and the *δ* ^13^C_VPDB‐LSVEC_ value of the generated CO_2_ was determined by a Thermo Fisher Scientific Delta V isotope‐ratio mass spectrometer (Bremen, Germany) [29]. Determination of *δ* ^13^C_VPDB‐LSVEC_ values used two‐point normalization [35] with two isotopic reference materials having different isotopic compositions selected from the three RMs NBS 19 limestone, USGS61 caffeine, and USGS41a l‐glutamic acid (Table 1). Reference materials were interspersed among samples for calibration. Peak size was monitored to verify that no matrix effects occurred between the organic and inorganic reference materials, all of which—along with the samples—contained the same carbon mass. All carbon isotope values from IRMS measurements discussed in the text were measured on the VPDB‐LSVEC scale. The standard combined uncertainties for RMs are those published (last column of Table 1) except that the values for USGS61 caffeine and USGS65 glycine have been increased by 0.01‰ to be conservative, based on additional analyses of these materials.

**TABLE 1:** Carbon isotopic abundances of isotopic reference materials used for normalization and quality control in this investigation. [*U*_c_ (*k* = 1), assigned standard combined uncertainty. Ref, reference.]

ID	Material	Purpose	*δ* ^13^C_VPDB‐LSVEC_ (‰)	*u*_c_ (*k* = 1) (‰)	Ref
NBS 19	Limestone	Normalization	+1.95	0	Hut [22]
USGS61	Caffeine	Normalization	−35.05	0.05	Schimmelmann et al. [34]
USGS41a	l‐Glutamic acid	Normalization	+36.55	0.08	Qi et al. [27]
USGS65	Glycine	Quality control	−20.29	0.05	Schimmelmann et al. [34]
USGS44	Calcium carbonate	Quality control	−42.21	0.05	Qi et al. [29]

### 2.3 NMR Data Collection

Analyses were performed using three different NMR spectrometers, representing each of three major manufacturers: (1) a Varian VNMRS 600 MHz spectrometer equipped with a broadband 5‐mm AutoXDB PFG probe with ^1^H coil external to the ^13^C coil; (2) a Bruker AvanceNEO 400 MHz instrument with a multinuclear probe; (3) a JEOL ECZL400S 400 MHz instrument with a multinuclear broadband probe. NMR samples contained 0.7 mL ^2^H_2_O, 2.4 to 4 mg of calcium formate reference material, 2 to 3 mg of USGS66 glycine reference material, and 4 mg of a 2,2,2‐trifluorethanol (TFE) in‐house standard (Thermo Scientific lot U03I023). Chromium (III) acetylacetonate (0.001 mL) was added to each NMR sample to reduce the T_1_ relaxation time of the measured ^1^H nuclei to 2 to 2.2 s. The ^1^H NMR spectrum of the formate does not overlap with the spectra of the USGS glycine and TFE, which were previously well‐characterized by IRMS [34, 36] and ^1^H NMR [14], so these molecules can serve as internal controls without interfering with the calcium formate analysis. NMR spectra were acquired at 25°C using a 90° ^1^H pulse followed by an acquisition time of 4.7 to 6 s, with 25 to 35 s between scans, which is greater than 10 times the longest T_1_ relaxation time of the detected ^1^H nuclei. Spectra were obtained without sample spinning to avoid spinning sidebands.

Spectra obtained using the 600‐MHz instrument were acquired in nine overnight datasets of 14 to 20 h each, three for each calcium formate reference material. Each of these nine datasets consisted of a “stack” of 144 to 300 consecutively acquired individual ^1^H spectra of 8 to 16 scans each (Figure S1). Spectra obtained using the 400‐MHz instruments were averages of 192 to 1536 individual scans, with total acquisition times of 2 to 15 h.

### 2.4 NMR Data Processing

Raw NMR data were processed using the MestReNova software [37] for apodization, zero filling, Fourier transformation, and phase correction. Each free induction decay (FID) was zero‐filled to two million points and multiplied by a decaying exponential and shifted sine‐squared function to produce a relatively flat baseline at the ^1^H‐^13^C satellite peaks and minimize truncation artifacts. The spectral region containing the central (^1^H‐^12^C) and satellite (^1^H‐^13^C) peaks was then exported from MestReNova in CSV format. An R script written in R (version 4.6.0) [38] was used to extract the region of the spectrum containing the central ^1^H‐^12^C and satellite ^1^H‐^13^C peaks. The satellite peaks (with shapes described by I_2_ and I_3_ in Equation 4) were aligned, summed, and digitally superimposed onto the corresponding central ^1^H‐^12^C peak (with shape described by I_1_ in Equation 4). Four variables were used to obtain the best fit to Equation (4): *R*(^13^C/^12^C) (*Ratio* in Equation (4)), a line broadening correction (*LB*), and corrections for baseline offset, slope, and curvature (*b*0, *b*1, and *b*2):

(4) $$\left(I_{2}+I_{3}\right)=\left(I_{1}\times \mathrm{LB}\times \text{Ratio}\right)+b0+b1\times x+b2\times x^{2}$$

*LB* is a Lorentzian line broadening factor that is used to match the widths of the central (^1^H‐^12^C) and satellite (^1^H‐^13^C) peaks; this accounts for the slight broadening of the satellite peaks due to the additional relaxation pathway that is introduced by the ^13^C nucleus. The term (+ *b*2 × *x* ^2^) accounts for the small amount of observed baseline curvature, and *x* is the point number along the frequency axis in the digitized spectrum. The fitting was carried out using the nonlinear least squares Levenberg–Marquardt (nlsLM) algorithm in R. Additional details of the NMR data processing are available in the Supporting Information, along with all raw NMR data and the R script used in processing each spectrum.

Our approach to determining *R*(^13^C/^12^C) in VPDB is as follows. The *δ* ^13^C values determined by IRMS on the VPDB–LSVEC scale were transformed to the VPDB scale using Equation (1). The transformed *δ* ^13^C values were then combined with the absolute ^13^C/^12^C ratios determined by NMR, and Equation (3) was rearranged to calculate *R*(^13^C/^12^C) in VPDB independently for each calcium formate material. We have chosen our approach because it directly reports *R*(^13^C/^12^C) in VPDB while preserving traceability to the VPDB–LSVEC‐normalized IRMS measurements.

## 3 Results

### 3.1 IRMS Results

The *δ* ^13^C_VPDB‐LSVEC_ values of all materials analyzed in this study are shown in Table 2. The calibration and isotopic homogeneity evaluation of USGS106, USGS107, and USGS108 were performed simultaneously. From the bulk material, we randomly selected three units of USGS106, two units of USGS107 and 11 units of USGS108 and analyzed three to five 0.4‐mg aliquots from each unit. Because USGS108 exhibited the highest level of ^13^C enrichment, 11 aliquots were evaluated to ensure homogeneity. For each of the analysis sequences, the RMs NBS 19, USGS61, and USGS41a were used for normalization. Analyzed in the same analytical runs were USGS65 glycine and USGS44 high‐purity calcium carbonate, which served as quality control materials. We calculated standard combined uncertainties, *u*_c_ (*k* = 1), of *δ* ^13^C_VPDB‐LSVEC_ values by combining repeat measurements (Type A uncertainty) and estimated instrumental and sample uncertainty (Type B uncertainty, based on our experience with repeat analyses of the same sample) using the square root of the sum of the square's relation. We included uncertainties of RMs used for normalization. The *δ* ^13^C_VPDB‐LSVEC_ values of USGS106, USGS107, and USGS108 were found to be −28.54‰ ± 0.05‰, −10.13‰ ± 0.03‰, and +13.84‰ ± 0.03‰, respectively (Table 2). A combined standard uncertainty of 0.05‰ or better from these measurements indicates that these calcium formate RMs are isotopically homogeneous to within 0.05‰ at 0.4‐mg level. The *δ* ^13^C_VPDB‐LSVEC_ value of the quality assurance RM USGS44 high‐purity calcium carbonate was −42.18‰ ± 0.05‰ (Table 2), which compares well with the published value [29] of −42.21‰ ± 0.05‰ (Table 1) and an estimated, unified value [31] of −42.25‰ ± 0.10‰. The *δ* ^13^C_VPDB‐LSVEC_ value of the quality assurance RM USGS65 glycine was −20.32‰ ± 0.05‰ (Table 2), which compares well with the published value of −20.29‰ ± 0.05‰ (Table 1).

**TABLE 2:** Measured *δ* ^13^C_VPDB‐LSVEC_ values and calculated *δ* ^13^C_VPDB_ values of materials analyzed in this study. [*u*_c_ (*k* = 1), measured combined standard uncertainty; *n*, number of measurements.]

ID	Material	Purpose	*δ* ^13^C_VPDB‐LSVEC_ (‰)	Measured *u*_c_ (*k* = 1) (‰)	*n*	*δ* ^13^C_VPDB_ ‰
NBS 19	Limestone	Normalization	+1.95	0.01	20	+1.95
USGS61	Caffeine	Normalization	−35.05	0.05	10	−34.95
USGS41a	l‐Glutamic acid	Normalization	+36.55	0.06	10	+36.45
USGS65	Glycine	Quality control	−20.32	0.05	2	−20.26
USGS44	Calcium carbonate	Quality control	−42.18	0.05	8	−42.06
USGS106	Calcium formate	To be calibrated	−28.54	0.05	15	−28.45
USGS107	Calcium Formate	To be calibrated	−10.13	0.03	10	−10.10
USGS108	Calcium formate	To be calibrated	+13.84	0.03	35	+13.81

### 3.2 NMR Results

Stacks of 144 to 300 consecutively recorded 600 MHz ^1^H NMR spectra of 8 to 16 scans each were used to determine the *R*(^13^C/^12^C) ratio at the single carbon position in each of the three calcium formate materials (Table 3). Acquiring the data in a stacked manner created opportunities to enhance the quality of the spectra: Each spectrum in the stack was individually phase‐corrected and aligned using an R script prior to summation, thereby compensating for magnetic field drift and phase drift, resulting in slightly sharper line widths and slightly improved signal‐to‐noise (Figure S2). Outlier spectra containing subtle artifacts were identified through a leave‐one‐out procedure, in which the peak‐shape fitting was repeated while sequentially omitting one spectrum at a time from the stack. We found that omitting up to 2% of the spectra from some of the data stacks meaningfully improved the shape fit and reduced the standard error of the *R* ^13^C/^12^C ratio term in the nonlinear least squares fit to Equation (2). This outlier rejection approach prevented transient vibrations or instrumental glitches from significantly degrading the quality of a 14‐ to 20‐h data acquisition.

**TABLE 3:** Summary 600 MHz ^1^H NMR results and *R*(^13^C/^12^C) ratios for USGS106, USGS107, and USGS108 calcium formate isotopic reference materials and for VPDB.

ID	Description	No. of NMR spectra	*R*(^13^C/^12^C)	*R*(^13^C/^12^C)_VPDB_
USGS106	^1^H NMR stack 1	300	0.0107871 ± 0.0000046	
USGS106	^1^H NMR stack 2	228	0.0107917 ± 0.0000054	
USGS106	^1^H NMR stack 3	180	0.0107878 ± 0.0000053	
	Weighted mean		0.0107888 ± 0.0000033	0.0111058 ± 0.0000034
USGS107	^1^H NMR stack 4	300	0.0109903 ± 0.0000057	
USGS107	^1^H NMR stack 5	220	0.0109936 ± 0.0000062	
USGS107	^1^H NMR stack 6	144	0.0109978 ± 0.0000060	
	Weighted mean		0.0109939 ± 0.0000048	0.0111061 ± 0.0000049
USGS108	^1^H NMR stack 7	171	0.0112540 ± 0.0000056	
USGS108	^1^H NMR stack 8	240	0.0112582 ± 0.0000057	
USGS108	^1^H NMR stack 9	238	0.0112625 ± 0.0000054	
	Weighted mean		0.0112583 ± 0.0000044	0.0111049 ± 0.0000044

[*R*(^13^C/^12^C) uncertainties are combined standard uncertainties (*k* = 1). *R*(^13^C/^12^C) values are derived from the 600‐MHz NMR spectra. *R*(^13^C/^12^C)_VPDB_ values were calculated using Equation (3) and mean *δ* ^13^C_VPDB_ values of USGS106, USGS107, and USGS108 in Table 2; uncertainties are standard combined uncertainties (*k* = 1).]

**FIGURE 1:**
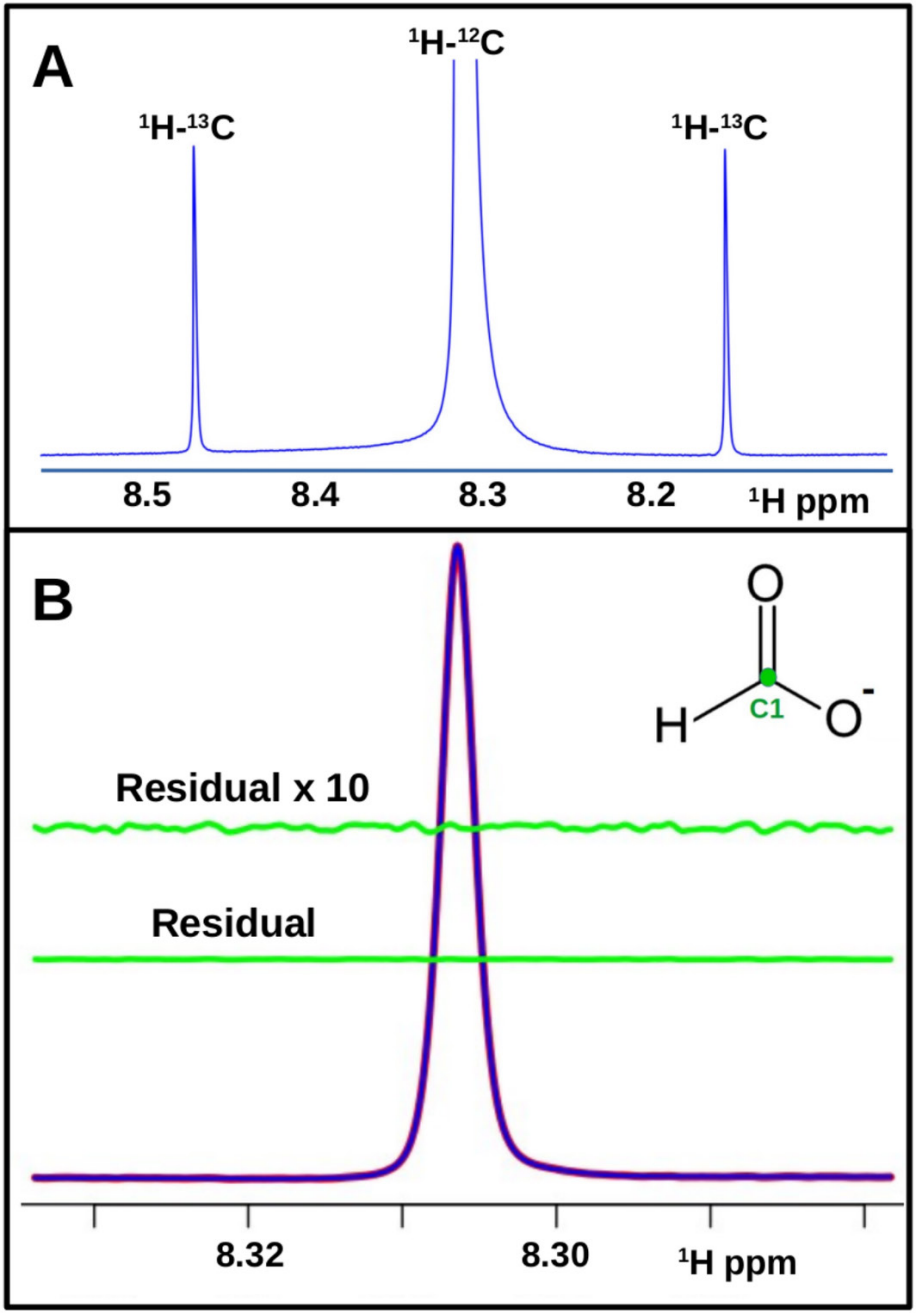
Illustration of the ^1^H NMR method for determining *R*(^13^C/^12^C). (A) Section of a 600‐MHz ^1^H NMR spectrum showing the central ^1^H‐^12^C and satellite ^1^H‐^13^C signals from formate. The *x*‐axis is NMR chemical shift. (B) The sum of the two ^1^H‐^13^C satellite peaks (red) superimposed onto the central ^1^H‐^12^C peak (blue) fit using Equation (4). The *R*(^13^C/^12^C) ratio is the scale factor (*Ratio* in Equation 4) for the superposition. The fit is close enough so that the separate red and blue lines are not separately visible; the residual (difference) between the red and blue lines is shown in green, along with the residual times 10 offset from the baseline for clarity.

Spectra obtained using JEOL 400 MHz and Bruker 400 MHz spectrometers yielded the same ^13^C/^12^C ratios for each calcium formate, within the experimental uncertainty (Figures S4 and S5). This lack of instrument dependence is consistent with our previous observations [14] and can be attributed to the data being acquired in a Fourier transform mode, where signals from ^1^H bound to ^12^C and ^1^H bound to ^13^C pass simultaneously through the same hardware before being separated by the Fourier transform operation. Thus, instrument imperfections such as magnetic field inhomogeneity and phase drift affect ^1^H‐^13^C and ^1^H‐^12^C signals equally and cancel out upon calculating the ^13^C/^12^C ratio [14]. The 600‐MHz spectra exhibit the most favorable signal‐to‐noise (Figure 1) and have a relatively low standard deviation between the acquired spectra (Table 3).

### 3.3 R(^13^C/^12^C) in the Three Calcium Formates

For each calcium formate material, individual spectra within a stack were summed to produce high‐quality spectra for analysis. The R(^13^C/^12^C) ratio was determined by finding the scale factor needed to superimpose the central ^1^H‐^12^C peak onto the sum of the two ^1^H‐^13^C satellite peaks (Figure 1). This approach exploits the similarity of the central and satellite peak shapes, and it allows us to avoid integrating the peaks, which is inaccurate due to the peak tails. It avoids having to fit an idealized shape (such as Gaussian and Lorentzian functions), which is problematic because the peaks are slightly asymmetric due to magnetic field inhomogeneity. The peak shape fitting procedure is insensitive to magnetic field inhomogeneity and small phase errors because these have an equal effect on the central and satellite peaks [14].

An apodization function was used to multiply the time‐domain NMR signal before Fourier transformation to produce a relatively flat baseline at the peaks of interest. The *R*(^13^C/^12^C) ratios obtained from the NMR data were not sensitive to the precise choice of apodization function that was used before Fourier transformation if the resulting baseline curvature was small. Similarly, the *R*(^13^C/^12^C) ratios were not influenced by the presence of additives (USGS66 glycine, TFE, and chromium acetylacetonate) because these had NMR peaks that are well separated from the formate signal. This was confirmed using additional data stacks collected without these internal standards and the chromium relaxation agent.

The uncertainty in the *R*(^13^C/^12^C) ratio was assessed by recalculating the ratio using subsets of each data stack. Subsets were created by omitting 25% of the spectra in each stack; the *R*(^13^C/^12^C) ratio was then calculated for each subset. Additional subsets of the data were generated by using different spectral widths (containing different numbers of points) to capture the shape of each peak, so that the *R*(^13^C/^12^C) ratio was calculated using different amounts of the peak tails (Figure S3). Finally, for each formate material, data were collected in three separate overnight NMR runs, in a total of nine overnight data collections. The *R*(^13^C/^12^C) ratios in the formate materials were determined using a weighted mean, where each data stack was weighted by the total number of scans used to produce the stack. For each of the three materials, the between‐run scatter (representing Type B uncertainty) in the *R*(^13^C/^12^C) ratios was higher than the within‐run (Type A) scatter; the uncertainty in the *R*(^13^C/^12^C) ratio for each material was inflated to account for the type B component. Results are summarized in Table 3, with the raw data and R scripts used for processing the NMR data available in the Supporting Information. The *R*(^13^C/^12^C) ratios and standard combined uncertainties (*k* = 1) obtained for formate materials USGS106, USGS107, and USGS108 were found to be 0.0107888 ± 0.0000033, 0.0109939 ± 0.0000048, and 0.0112583 ± 0.0000044, respectively.

### 3.4 R(^13^C/^12^C) Ratio in VPDB

The *δ* ^13^C_VPDB_ values computed using Equation (1) were compared with the *R*(^13^C/^12^C) ratios from the ^1^H NMR spectra to determine the ratio of *R*(^13^C/^12^C) of VPDB (*R*(^13^C/^12^C)_VPDB_) that best intercalibrates the IRMS and NMR results (Table 3). We calculated the standard combined uncertainties of *R*(^13^C/^12^C)_VPDB_ values (Table 3) by combining the standard combined uncertainties of *δ* ^13^C_VPDB‐LSVEC_ values and the uncertainties of *R*(^13^C/^12^C) measurements by 600 MHz NMR. The uncertainties in *R*(^13^C/^12^C)_VPDB_ values are due almost entirely to the uncertainties of NMR measurements (refer to Supporting Information). The values of *R*(^13^C/^12^C)_VPDB_ derived from each of the three reference materials were fully consistent within the uncertainties. The value with expanded combined uncertainty of *R*(^13^C/^12^C)_VPDB_ derived from the nine NMR data stacks and all IRMS results was found to be 0.0111050 ± 0.0000047 (*k* = 2). Note that the value 0.0111050 is a weighted mean, weighted by the uncertainties of the contributing components.

## 4 Discussion

The calcium formate reference materials were selected based on their suitability for use as standards in the analysis of carbon stable isotope abundance using either IRMS or ^1^H NMR. These materials can be particularly advantageous as internal standards for *R*(^13^C/^12^C) analysis by ^1^H NMR for three reasons: (1) Their ^1^H NMR spectra show only a single sharp peak with a pair of ^1^H–^13^C satellites located near 8.3 ppm, at positions that generally do not overlap with signals from other organic compounds; (2) they are free of detectable organic impurities, as confirmed by ^1^H NMR; and (3) both bulk and position‐specific *R*(^13^C/^12^C) abundances have now been determined with high accuracy.

In contrast, previously available reference materials such as glycines USGS64, USGS65, USGS66 [34], and LGC171‐KT [39] lacked these advantages. Their ^1^H NMR signals (near 3.4 ppm) overlapped with those of many biological compounds, including amino acids and nucleotides; they contain two carbon positions [14], complicating direct comparisons with IRMS results (which report average *δ* ^13^C values) with ^1^H NMR results (which provide position‐specific *R*(^13^C/^12^C) ratios). The new calcium formate standards therefore provide distinct advantages as reference materials for ^1^H NMR studies of position‐specific *R*(^13^C/^12^C) ratios.

The use of isotope standards is a routine procedure in IRMS and can also be used when NMR methods are applied to measure *R*(^13^C/^12^C) ratios in unknown samples. Internal standards such as these calcium formate reference materials can be used for control of instrument stability and detect possible nonlinearity in the response of the NMR instrumentation, such as may be introduced by radiation damping.

Limitations of calcium formate as an NMR standard include its relatively long ^1^H T_1_ NMR relaxation time of ~9 s, which leads to long delays between NMR scans for signal averaging, and long data acquisition times when quantitative analysis is performed. However, we found that this disadvantage can be eliminated by adding a small amount of chromium acetylacetonate to the NMR sample, which reduces the T_1_ relaxation time to a more favorable ~2 s without introducing significant line broadening or large additional peaks into the NMR spectrum, allowing for more rapid data acquisition. We also note that calcium formate is not soluble in nonpolar NMR solvents. Future development of nonpolar reference materials for ^1^H NMR studies of *R*(^13^C/^12^C) measurements could be beneficial.

**FIGURE 2:**
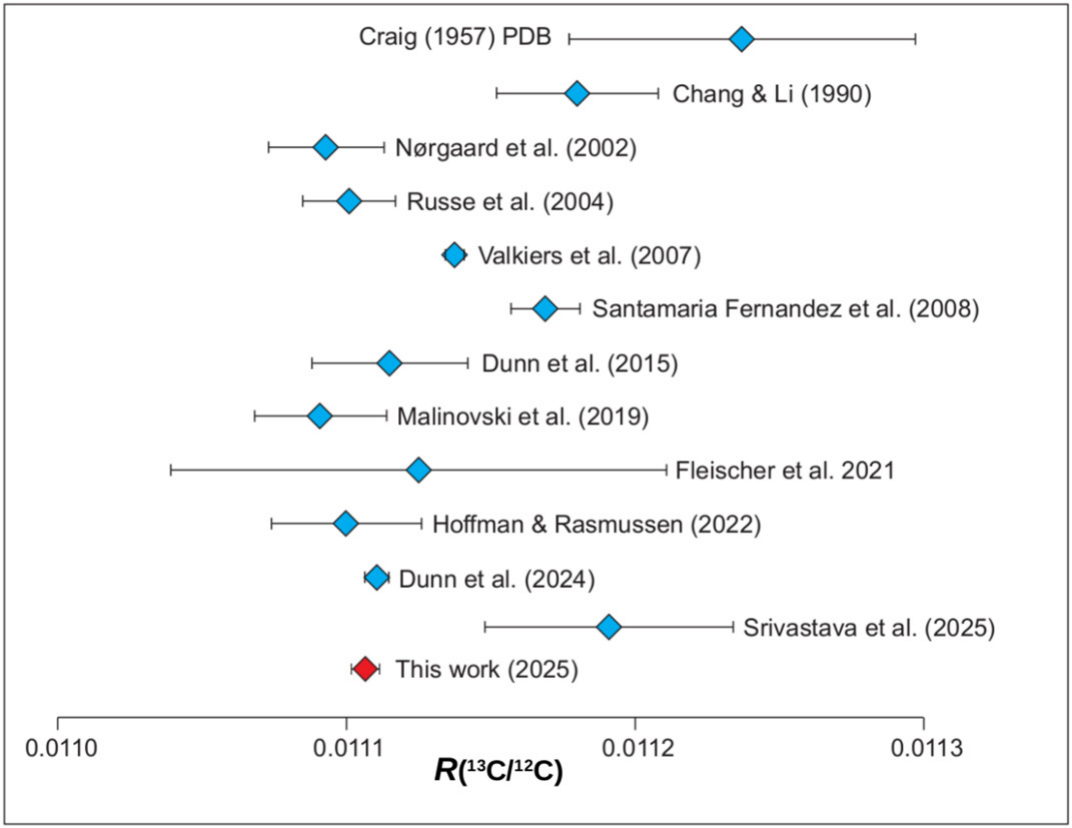
Summary of historical values for the *R*(^13^C/^12^C) ratio in VPDB in comparison with the present study. Error bars represent the expanded uncertainty (*k* = 2, level of confidence 95%). References are Craig [41], Chang and Li [42], Nørgaard et al. [43], Russe et al. [44], Valkiers et al. [45], Santamaria‐Fernandez et al. [46], Dunn et al. [47], Malinovski et al. [39], Fleischer et al. [18], Hoffman and Rasmussen [14], Dunn et al. [40], and Srivastava et al. [19].

The value of 0.0111050 ± 0.0000047 (*k* = 2) obtained for the *R*(^13^C/^12^C) in VPDB in the present work agrees with the recent high‐precision determination of 0.0111105 ± 0.0000042 (*k* = 2) by Dunn et al. [40], which was achieved using entirely different analytical methods (Figure 2). Our results are also consistent with the CIAAW‐recommended value [28] of 0.011113 ± 0.000029 and the value of 0.011100 ± 0.000026 that we reported previously using ^1^H NMR [14]. However, the present work's use of a more ideal RM, an improved NMR data acquisition strategy, and more precise IRMS measurements enabled achievement of an approximately fivefold lower uncertainty.

Together, the present ^1^H NMR and IRMS measurements contribute to an improved understanding of the *R*(^13^C/^12^C) ratio in VPDB. After decades of effort, the value of *R*(^13^C/^12^C)_VPDB_ has now become much better constrained (Figure 2). Our determination of the *R*(^13^C/^12^C) value for VPDB and the high precision determination by Dunn et al. [40] agree within their experimental uncertainties, and each differs substantially (by over 6‰) from the widely used value [42, 48] of 0.011180. This provides evidence that the *R*(^13^C/^12^C) ratio in VPDB has been incorrectly estimated in past decades.

Many geochemical and biogeochemical studies draw their conclusions from relative differences rather than measurements of *R*(^13^C/^12^C) values and are therefore unaffected by revisions in the *R*(^13^C/^12^C) value for VPDB. However, an accurate value for *R*(^13^C/^12^C) in VPDB is essential for inter‐method comparisons. For example, techniques such as ^1^H NMR, ^19^F NMR, ^31^P NMR, and FTIR yield isotope ratios, not relative isotope ratios, and they depend on an accurate value for the *R*(^13^C/^12^C) ratio in VPDB for comparison with IRMS‐based studies.

## 5 Conclusion

There has been a lack of suitable reference materials for intercalibrating *R*(^13^C/^12^C) ratios measured by ^1^H NMR with *δ* ^13^C values obtained by IRMS. To address this need, we developed and characterized three calcium formate reference materials suitable for accurate measurement by either ^1^H NMR spectroscopy or IRMS. For each material, we report both the *R*(^13^C/^12^C) ratio determined by ^1^H NMR, the relative isotope abundance (*δ* ^13^C_VPDB‐LSVEC_) determined by IRMS, and the calculated *δ* ^13^C_VPDB_ value.

Using the combined NMR and IRMS results, we calculated the *R*(^13^C/^12^C) ratio of the virtual Vienna Peedee belemnite as 0.0111050 ± 0.0000047 (*k* = 2), where the uncertainty is an expanded combined uncertainty. With the availability of these reference materials and a more firmly established *R*(^13^C/^12^C) value in VPDB, *R*(^13^C/^12^C) results from ^1^H NMR can now be reliably compared with *δ* ^13^C_VPDB‐LSVEC_ values from IRMS, improving the consistency of carbon isotope ratio measurements across analytical approaches.

The calcium formate reference materials USGS106, USGS107, and USGS108 are distributed by the USGS and can serve as valuable tools for method validation, instrument calibration, and interlaboratory comparisons in carbon stable isotope ratio research.

## Author Contributions

D.H. and C.R. performed the NMR analyses and contributed to writing the manuscript. A.S. prepared the fine‐grained original and spiked calcium formates and sealed long‐term supplies under vacuum in round‐bottom glass flasks. L.T.R. performed IRMS analyses and review. H.Q. evaluated materials homogeneity and isotope analyses obtained by IRMS and contribute to writing. T. B. Coplen contributed to writing – review and editing.

## Conflicts of Interest

The authors declare no conflicts of interest.

## Supporting information




**FIGURE S1:** Example of stacked spectra for *R*(^13^C/^12^C) analysis of calcium formate. Sections of eight ^1^H spectra containing the formate peaks are shown from a stack of 300 consecutively acquired 600‐MHz spectra. The sample contained 2.5 mg of calcium formate material USGS106 (unspiked calcium formate), 3.0 mg of the USGS66 glycine reference material, 4 mg 2,2,2‐trifluorethanol (TFE) in‐house standard, 1 μL of chromium acetylacetonate (from a saturated solution in ^2^H_2_O), and ^2^H_2_O up to a total volume of 0.7 mL. Spectra were processed by multiplying the free induction decay by a shifted sine function and a decaying exponential function prior to Fourier transformation. The spectra were aligned and summed (see Figure S2) prior to determination of the *R*(^13^C/^12^C) ratio in the formate, by superimposing the sum of the ^1^H‐^13^C peaks onto the central ^1^H‐^12^C peak using an R script for a nonlinear least squares shape fit. Raw data file = caf_regular_01.fid.
**FIGURE S2:** Example of spectrum alignment prior to *R*(^13^C/^12^C) analysis. Sections of eight ^1^H spectra of the formate material USGS108 peaks are shown, superimposed. The eight spectra were selected from within a stack of 238 consecutively acquired 600‐MHz spectra to show the full range of offsets in the full stacked data set. Alignment of the spectra compensates for field drift during the 14‐h data collection and results in a slightly reduced line width and improved signal‐to‐noise ratio when the spectra in the stack are summed. All spectra were multiplied by the same apodization function (shifted sine squared and 0.3 Hz line broadening) prior to Fourier transformation. The raw data file is caf_heavy_03.fid.
**FIGURE S3:** Example showing that *R*(13C/12C) abundance in material USGS107 determined by ^1^H NMR does not strongly depend on the number of points used to define the central and satellite peak shapes in the superposition procedure. (A) Using 6000 points and a narrow frequency window to define the peak shapes, the sum of the two ^1^H‐^13^C satellite peaks (red) is superimposed onto the central ^1^H‐^12^C peak (blue) fit using Equation (2). The fit is sufficiently close so that the red and blue lines are not separately visible; the residual (difference) between the red and blue lines is shown in green, along with the residual times 10 in purple, offset from the baseline for clarity. (B) Using 10 000 points and a wide frequency window to define the peak shapes, the sum of the two ^1^H‐^13^C satellite peaks (red) is superimposed onto the central ^1^H‐^12^C peak (blue). (C) Plot showing that the *R*(^13^C/^12^C) abundance determined by NMR does not depend on the number of points used to define the peak shapes. For the figure, the *R*(^13^C/^12^C) ratio of USGS107 from NMR was converted to *δ* ^13^C_VPDB_ using 0.0111050 for the ratio of *R*(^13^C/^12^C) in VPDB. The raw data file name is caf_medium_03.fid.
**FIGURE S4:** Example of an NMR analysis to determine the *R*(^13^C/^12^C) ratio in USGS108 calcium formate using a JEOL 400‐MHz spectrometer. Exactly 1536 scans were averaged to produce the spectrum. (A) Section of the ^1^H NMR spectrum showing the central ^1^H‐^12^C and satellite ^1^H‐^13^C peaks. (B) The sum of the two ^1^H‐^13^C satellite peaks (blue) is superimposed on the central ^1^H‐^12^C peak (red). The *R*(^13^C/^12^C) ratio is the scale factor (*ratio* in eq. 4) for the superposition. The fit is sufficiently close so that the red and blue lines are not separately visible; the residual (difference) between the red and blue lines is shown in green, along with the residual times 10 in purple, offset from the baseline for clarity. This NMR analysis using the 400‐MHz data produced a *R*(^13^C/^12^C) ratio of 0.011258(5) for the USGS108 calcium formate, which is consistent with the 600‐MHz data, within the experimental uncertainty. The raw data file is caf_heavy_jeol_01.
**FIGURE S5:** Example of an NMR analysis to determine the *R*(^13^C/^12^C) ratio in USGS108 calcium formate using a Bruker 400 MHz spectrometer. Exactly 192 scans were averaged to produce the spectrum. (A) Section of the ^1^H NMR spectrum showing the central ^1^H‐^12^C and satellite ^1^H‐^13^C peaks. (B) The sum of the two ^1^H‐^13^C satellite peaks (blue) is superimposed on the central ^1^H‐^12^C peak (red). The *R*(^13^C/^12^C) ratio is the scale factor for the superposition. The fit is sufficiently close so that the red and blue lines are not separately visible; the residual (difference) between the red and blue lines is shown in green, along with the residual times 10 in purple, offset from the baseline for clarity. This NMR analysis using the 400‐MHz NMR data produced a *R*(^13^C/^12^C) ratio of 0.011264(10) for USGS108 calcium formate, which is consistent with the 600‐MHz data, within the experimental uncertainties. The raw data file is caf_heavy_neo400_02.

## Data Availability

The data that support the findings of this study are openly available in Texas Data Repository at https://doi.org/10.18738/T8/2JOLKX.
