# Supplementary material for: Three New Calcium Formate Reference Materials for δ 13C Measurements and a Redetermination of the R(13C/12C) Ratio for VPDB Based on Proton Nuclear Magnetic Resonance Measurements
Source: Rapid Commun Mass Spectrom. 2026 Jul 1;40(18):e70132. doi: 10.1002/rcm.70132 (PMC13320617; doi:10.1002/rcm.70132)
Supplement: Supplementary file 1 — FIGURE S1: Example of stacked spectra for R(13C/12C) analysis of calcium formate. Sections of eight 1H spectra containing the formate peaks are shown from a stack of 300 consecutively acquired 600‐MHz spectra. The sample contained 2.5 mg of calcium formate material USGS106 (unspiked calcium formate), 3.0 mg of the USGS66 glycine reference material, 4 mg 2,2,2‐trifluorethanol (TFE) in‐house standard, 1 μL of chromium acetylacetonate (from a saturated solution in 2H2O), and 2H2O up to a total volume of 0.7 mL. Spectra were processed by multiplying the free induction decay by a shifted sine function and a decaying exponential function prior to Fourier transformation. The spectra were aligned and summed (see Figure S2) prior to determination of the R(13C/12C) ratio in the formate, by superimposing the sum of the 1H‐13C peaks onto the central 1H‐12C peak using an R script for a nonlinear least squares shape fit. Raw data file = caf_regular_01.fid. FIGURE S2: Example of spectrum alignment prior to R(13C/12C) analysis. Sections of eight 1H spectra of the formate material USGS108 peaks are shown, superimposed. The eight spectra were selected from within a stack of 238 consecutively acquired 600‐MHz spectra to show the full range of offsets in the full stacked data set. Alignment of the spectra compensates for field drift during the 14‐h data collection and results in a slightly reduced line width and improved signal‐to‐noise ratio when the spectra in the stack are summed. All spectra were multiplied by the same apodization function (shifted sine squared and 0.3 Hz line broadening) prior to Fourier transformation. The raw data file is caf_heavy_03.fid. FIGURE S3: Example showing that R(13C/12C) abundance in material USGS107 determined by 1H NMR does not strongly depend on the number of points used to define the central and satellite peak shapes in the superposition procedure. (A) Using 6000 points and a narrow frequency window to define the peak shapes, the sum of the two 1H [file RCM-40-e70132-s001.docx]

**Supporting Information**

**for**

**Three new calcium formate reference materials for *δ*^13^C measurements and a redetermination of the *R*(¹³C/¹²C) ratio for VPDB based on proton nuclear magnetic resonance measurements**

David W. Hoffman^1^*, Cornelia Rasmussen^2^, Arndt Schimmelmann^3^, Lauren T. Reid^4^,
Haiping Qi^4^, Tyler B. Coplen^4^

^1^Department of Molecular Biosciences, College of Natural Science, University of Texas at Austin, Austin, TX 78712, USA ([dhoffman@mail.utexas.edu](mailto:dhoffman@mail.utexas.edu))

^2^University of Texas Institute for Geophysics, Jackson School of Geosciences, University of Texas at Austin, Austin, TX 78758, USA

^3^Department of Earth and Atmospheric Sciences, Indiana University, Bloomington, Indiana 47405, USA

^4^U.S. Geological Survey, 431 National Center, Reston, Virginia 20192, USA

*Corresponding author

**Contents:**  **page**
Calculation of the combined mean and combined standard uncertainty in ^13^*R*_VPDB_ S-2

Example of stacked ^1^H spectra for *R*(^13^C/^12^C) analysis of formate (Fig. S1) S-6

Example of spectrum alignment prior to *R*(^13^C/^12^C) analysis (Fig. S2) S-7

*R*(^13^C/^12^C) versus the number of points used to define the peak shapes (Fig. S3) S-8

*R*(^13^C/^12^C) analysis using a 400-MHz JEOL spectrometer (Fig. S4) S-9

*R*(^13^C/^12^C) analysis using a 400-MHz Bruker spectrometer (Fig. S5) S-10

**NMR data** used in this study, along with the R scripts for determination of *R*(^13^C/^12^C) isotope ratios may be downloaded from the Texas Data Repository at: <https://doi.org/10.18738/T8/2JOLKX>

Any use of trade, firm, or product names is for descriptive purposes only and does not imply endorsement by the U.S. Government.

**Calculation of the** **combined mean and combined standard uncertainty in ^13^*R*_VPDB_**

For the three materials (USGS106, USGS107, USGS108), we have multiple measurements of *δ*^13^C_VPDB-LSVEC_ by isotope ratio mass spectrometry (IRMS), calculated values of *δ*^13^C_VPDB_, and multiple measurements of the ratio *R*(^13^C/^12^C) by NMR.

The IRMS-based values (*δ*^13^C_VPDB_) and NMR results, *R*(¹³C/¹²C)_NMR_, are used together to determine the *R*(^13^C/^12^C) ratio for VPDB (^13^*R*_VPDB_). The IRMS-based measurements and the NMR results are related by the equation:

*δ*^13^C_IRMS_ = ( *R*(¹³C/¹²C)_NMR_ / ^13^*R*_VPDB_ ) – 1 (1)

**Definitions and notes:** We follow the *Guide to the Expression of Uncertainty in Measurement* (GUM) protocols^1^ when combining the uncertainties from IRMS-based values and NMR measurements.

Type A uncertainty is a measure of scatter in repeated measurements; Type B uncertainty reflects uncertainty in the measurement system, estimated from knowledge and experience.

For IRMS-based results, *u*_c_ (*k* = 1) is the combined standard uncertainty, which includes both Type A and Type B uncertainties that arise from IRMS normalization. For NMR data, *u*_c_ (*k* = 1) is the combined standard uncertainty, including both Type A and Type B contributions. Type A is estimated from intra-stack standard deviations (typically 0.15 to 0.25 ‰) and Type B from inter-stack variation (typically 0.2 to 0.5 ‰). The uncertainty budget assumed that the calcium formate samples were homogeneous.

Small numbers are stated using exponential notation, to preserve clarity and reduce the chance of omitting zeroes during calculations. The *δ*^13^C_VPDB_ values are treated as dimensionless ratios.

**Step 1A:** For the **USGS106** samples, the IRMS-based results (*δ*^13^C_VPDB_) and NMR results *R*(¹³C/¹²C)_NMR_ are used together to find the *R*(^13^C/^12^C) ratio in VPDB (^13^*R*_VPDB_).

IRMS: *δ*^13^C_VPDB_ = –0.02845 = –28.45 ‰, *u*_c_ (*k* = 1) = 4.5E-05

NMR: *R*(¹³C/¹²C) = 0.0107888, *u*_c_ (*k* = 1) = 3.25E-06

The input values for Eq. (1) are:

*δ*^13^C_VPDB_ = –0.02845 *u*_c_ (*k* = 1) = 4.5E-05

*R*(¹³C/¹²C)_NMR_ = 0.0107888 *u*_c_ (*k* = 1) = 3.25E-06

These input values yield: ^13^*R*_VPDB_ = 0.01110473

**Step 1B:** The uncertainty in ^13^*R*_VPDB_ derived from material **USGS106** is calculated using the partial derivative method of propagation of uncertainty (GUM protocol). We assume that the IRMS and NMR results are independent.

*A* = *R*(¹³C/¹²C)_NMR_

*B* = 1 + *δ*^13^C_VPDB_

*R* = ^13^*R*_VPDB_ = *A*/*B*

The uncertainty of the value of ^13^*R*_VPDB_ is produced from the IRMS and NMR results by division; therefore, the partial derivatives yield:

(*u_R_*/*R*)^2^ = (*u_A_*/*A*)^2^ + (*u_B_*/*B*)^2^

NMR term: *u_A_*/*A* = 3.25E-06 / 0.0107888 = 3.01E-04

IRMS term: *u_B_*/*B* = *U_δ_* / (1 + *δ*^13^C) = 4.5E-05 / 0.97155 = 4.63E-05

Combine in quadrature: *U_R_*/*R* = sqrt[ (3.01E-04)^2^ + (4.63E-05)^2^ ] = 3.05E-04

*u_R_* = *R* × (3.05E-04) = (0.01110473) × (3.05E-04) = 3.4E-06

The final result for ^13^*R*_VPDB_ derived from **USGS106**:

^13^*R*_VPDB_ = 0.01110473 (*u_c_* = 3.4E-06, *k* = 1).

Or, ^13^*R*_VPDB_ = 0.01110473 (340) (Note: extra digits are carried here for traceability)

Or, ^13^*R*_VPDB_ = 0.01110473 + 0.00000340

We observe that 97.7 % of the uncertainty budget is from NMR measurements, and 2.3 % comes from IRMS measurements.

**Step 2A:** Using data obtained from the **USGS107** materials, we repeat the process of Step 1A.

IRMS: *δ*^13^C_VPDB_ = –0.01010 = –10.10 ‰, *u*_c_ (*k* = 1) = 2.5E-05

NMR: *R*(¹³C/¹²C) = 0.0109939, *u*_c_ (*k* = 1) = 4.8E-06

The input values for Eq. (1) are:

*δ*^13^C_VPDB_ = –0.01010 *u*_c_ (*k* = 1) = 2.5E-05

*R*(¹³C/¹²C)_NMR_ = 0.0109939 *u*_c_ (*k* = 1) = 4.8E-06

These input values yield: ^13^*R*_VPDB_ = 0.01110608

**Step 2B:** Calculation of the uncertainty in the ^13^*R*_VPDB_ value using results from material **USGS107** is calculated repeating the process used in Step 1B.

*A* = *R*(¹³C/¹²C)_NMR_

*B* = 1 + *δ*^13^C_VPDB_

*R* = ^13^*R*_VPDB_ = *A*/*B*

(*u_R_*/*R*)^2^ = (*u_A_*/*A*)^2^ + (*u_B_*/*B*)^2^

NMR term: *U_A_*/*A* = 4.8E-06 / 0.0109939 = 4.36E-04

IRMS term: *U_B_*/*B* = *U_δ_* / (1 + *δ*^13^C) = 2.5E-05 / 0.98990 = 2.53E-05

Combine in quadrature: *u_R_*/*R* = sqrt[ (4.36E-04)^2^ + (2.53E-05)^2^ ] = 4.37E-04

*u_R_* = *R* × (4.37E-04) = (0.01110608) × (4.37E-04) = 4.85E-06

The final result for ^13^*R*_VPDB_ derived from **USGS107**:

^13^*R*_VPDB_ = 0.01110608 (*u_c_* = 4.85E-06, *k* = 1).

Or, ^13^*R*_VPDB_ = 0.01110608 (485) (Note: extra digits are carried here for traceability)

Or, ^13^*R*_VPDB_ = 0.01110608 + 0.00000485

Similar to USGS106, the uncertainty budget for USGS107 is dominated by the NMR term, which accounts for approximately 99 % of the uncertainty.

**Step 3A:** For the **USGS108** material, we repeat the process of Step 2A.

IRMS: *δ*^13^C_VPDB_ = +0.01381 = +13.81 ‰, *u*_c_ (*k* = 1) = 3.2E-05

NMR: *R*(¹³C/¹²C) = 0.0112583, *u*_c_ (*k* = 1) = 4.42E-06

The input values for Eq. (1) are:

*δ*^13^C_VPDB_ = +0.01381 *u*_c_ (*k* = 1) = 3.2E-05

*R*(¹³C/¹²C)_NMR_ = 0.0112583 *u*_c_ (*k* = 1) = 4.42E-06

These input values yield: ^13^*R*_VPDB_ = 0.01110490

**Step 3B:** Calculation of uncertainty in ^13^*R*_VPDB_ using results for **USGS108**, repeating the process of Step 2B.

*A* = *R*(¹³C/¹²C)_NMR_ = *R*_nmr_

*B* = 1 + *δ*^13^C_VPDB_ = 1.01381

*R* = ^13^*R*_VPDB_ = *A*/*B*

(*u_R_*/*R*)^2^ = (*u_A_*/*A*)^2^ + (*u_B_*/*B*)^2^

NMR term: *u_A_*/*A* = 4.42E-06 / 0.0112583 = 3.93E-04

IRMS term: *u_B_*/*B* = *u_B_* / (1 + *δ*^13^C) = 3.2E-05 / 1.01381 = 3.16E-05

Combine in quadrature: *u_R_*/*R* = sqrt[ (3.93E-04)^2^ + (3.16E-05)^2^ ] = 3.94E-04

*u_R_* = *R* × (3.94E-04) = ( 0.01110490) × (3.94E-04) = 4.38E-06

The final result for ^13^*R*_VPDB_ derived from **USGS108**:

^13^*R*_VPDB_ = 0.01110490 (*u_c_* = 4.38E-06, *k* = 1).

Or, ^13^*R*_VPDB_ = 0.01110490 (438) (Note: extra digits are carried here for traceability)

Or, ^13^*R*_VPDB_ = 0.01110490 + 0.00000438

As for the other samples, the uncertainty budget for USGS108 is dominated by the NMR term, which contributes approximately 99 % of the total uncertainty.

**Step 4:** Calculate the combined mean and combined standard uncertainty in ^13^*R*_VPDB_ using results for USGS106, USGS107 and USGS108 together.

**USGS106:** ^13^*R*_VPDB_ = 0.01110473 (*u_c_* = 3.40E-06, *k* = 1)

**USGS107:** ^13^*R*_VPDB_ = 0.01110608 (*u_c_* = 4.85E-06, *k* = 1)

**USGS108:** ^13^*R*_VPDB_ = 0.01110490 (*u_c_* = 4.38E-06, *k* = 1)

weights: w_106_ = 8.65 E10

w_107_ = 4.25 E10

w_108_ = 5.21 E10

Weighted mean (with weighting by 1/*u*_c_^2^) = 0.0111050

Combined uncertainty, *u*_RC_ = 1/sqrt(sum(w_i_)) = 2.35E-06 (*k* = 1)

**Final result**: ^13^*R*_VPDB_ = 0.0111050 (24) (*k* = 1)

**Final result**: ^13^*R*_VPDB_ = 0.0111050 (47) (*k* = 2)

**Final result with expanded combined uncertainty: ^13^*R*_VPDB_ = 0.0111050 (47) (*k* = 2)**

**Or, ^13^*R*_VPDB_ = 0.0111050 + 0.0000047 (*k* = 2)**

**Consistency check**:

For each of three samples, three NMR stacks were acquired and analyzed. The nine NMR data stacks can be paired with their corresponding IRMS measurements to provide nine determinations of ^13^*R*_VPDB_. All nine determinations fall in the range of **0.0111007 to 0.0111100**, which is consistent with the stated mean value and *u*_c_ (*k* = 2) uncertainty of **0.0111050 (47)**.

Finally, the analyses of ^13^*R*_VPDB_ based on USGS106, USGS107 and USGS108 are consistent with each other. Thus, our final result for ^13^*R*_VPDB_ is not sensitive to choice of sample, or the weighting of each data set.


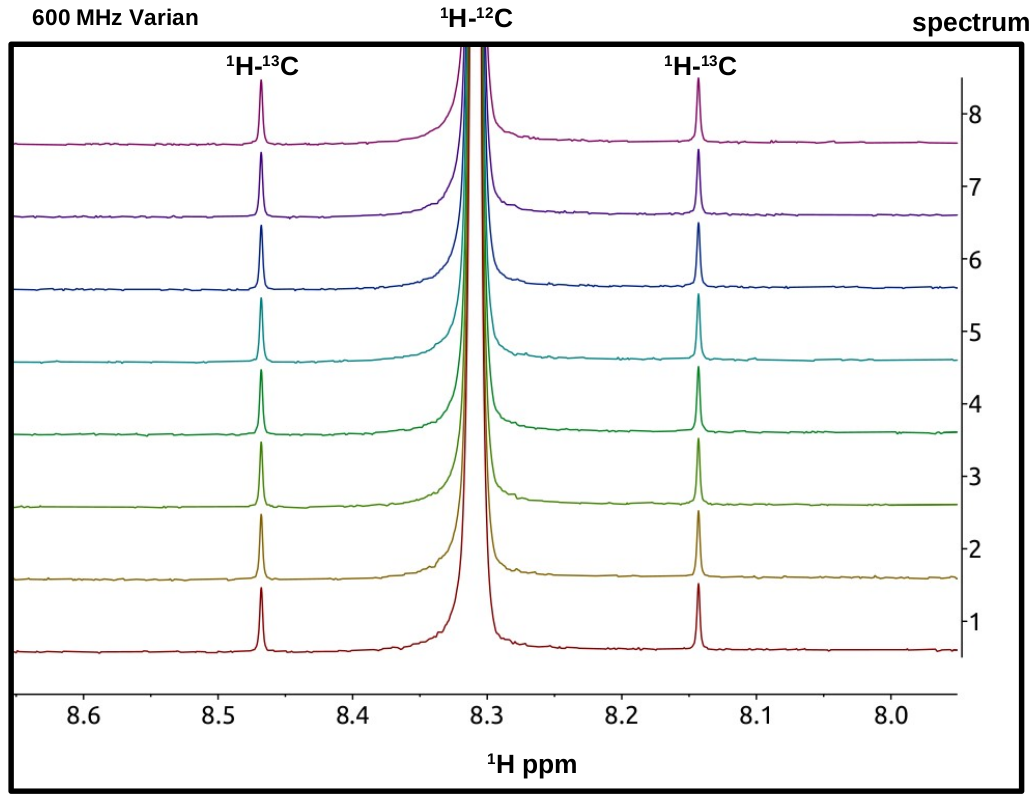


**Figure S1: Example of stacked spectra for *R*(^13^C/^12^C) analysis of calcium formate.** Sections of eight ^1^H spectra containing the formate peaks are shown from a stack of 300 consecutively acquired 600-MHz spectra. The sample contained 2.5 mg of calcium formate material USGS106 (unspiked calcium formate), 3.0 mg of the USGS66 glycine reference material, 4 mg 2,2,2-trifluorethanol (TFE) in-house standard, 1 microliter of chromium acetylacetonate (from a saturated solution in ^2^H_2_O) and ^2^H_2_O up to a total volume of 0.7 mL. Spectra were processed by multiplying the free induction decay by a shifted sine function and a decaying exponential function prior to Fourier transformation. The spectra were aligned and summed (see Fig. S2) prior to determination of the *R*(^13^C/^12^C) ratio in the formate, by superimposing the sum of the ^1^H-^13^C peaks onto the central ^1^H-^12^C peak using an R script for a non-linear least squares shape fit. Raw data file = caf_regular_01.fid.


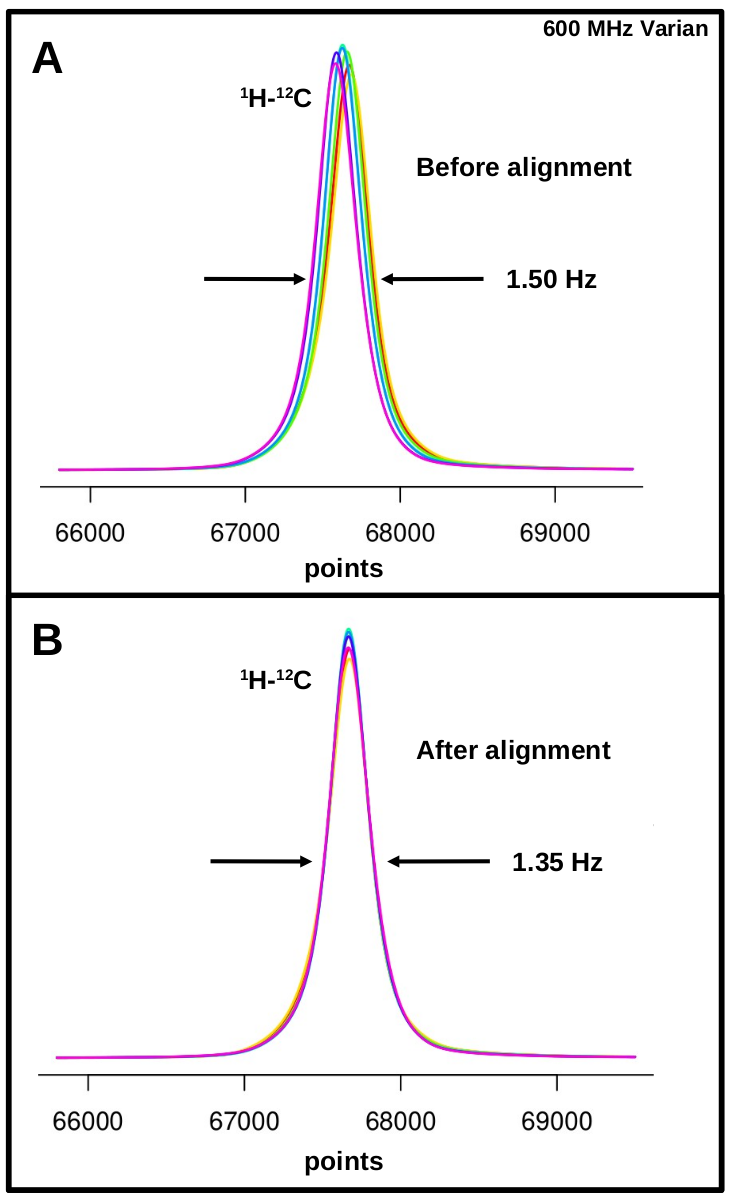


**Figure S2:** Example of spectrum alignment prior to *R*(^13^C/^12^C) analysis. Sections of eight ^1^H spectra of the formate material USGS108 peaks are shown, superimposed. The eight spectra were selected from within a stack of 238 consecutively acquired 600-MHz spectra to show the full range of offsets in the full stacked data set. Alignment of the spectra compensates for field drift during the 14-hour data collection and results in a slightly reduced line width and improved signal-to-noise ratio when the spectra in the stack are summed. All spectra were multiplied by the same apodization function (shifted sine squared and 0.3 Hz line broadening) prior to Fourier transformation. The raw data file is caf_heavy_03.fid.


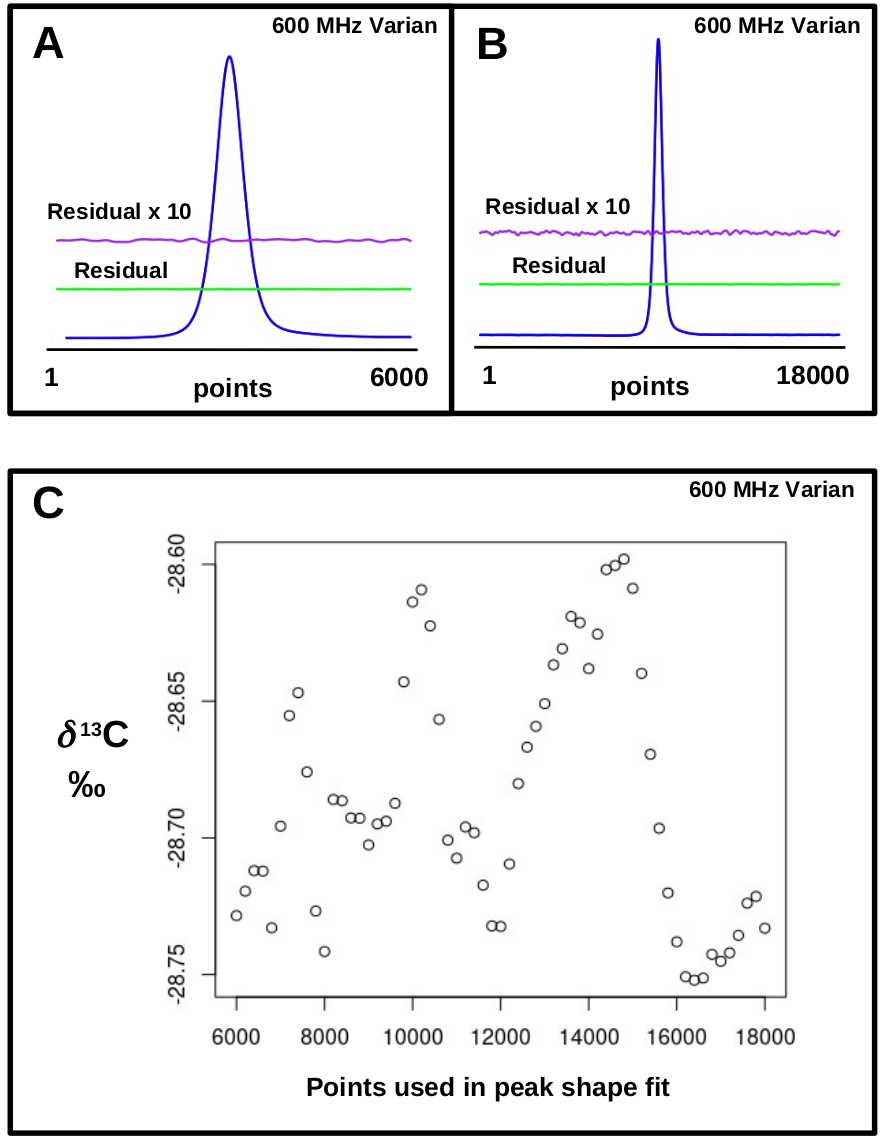


**Figure S3:** Example showing that *R*(^13^C/^12^C) abundance in material USGS107 determined by ¹H NMR does not strongly depend on the number of points used to define the central and satellite peak shapes in the superposition procedure. A) Using 6000 points and a narrow frequency window to define the peak shapes, the sum of the two ¹H-¹³C satellite peaks (red) is superimposed onto the central ¹H-¹²C peak (blue) fit using eq. 2. The fit is sufficiently close so that the red and blue lines are not separately visible; the residual (difference) between the red and blue lines is shown in green, along with the residual times ten in purple, offset from the baseline for clarity. B) Using 10,000 points and a wide frequency window to define the peak shapes, the sum of the two ¹H-¹³C satellite peaks (red) is superimposed onto the central ¹H-¹²C peak (blue). C) Plot showing that the *R*(^13^C/^12^C) abundance determined by NMR does not depend on the number of points used to define the peak shapes. For the figure, the *R*(¹³C/¹²C) ratio of USGS107 from NMR was converted to *δ*^13^C_VPDB_ using 0.0111050 for the ratio of *R*(¹³C/¹²C) in VPDB. The raw data file name is caf_medium_03.fid.


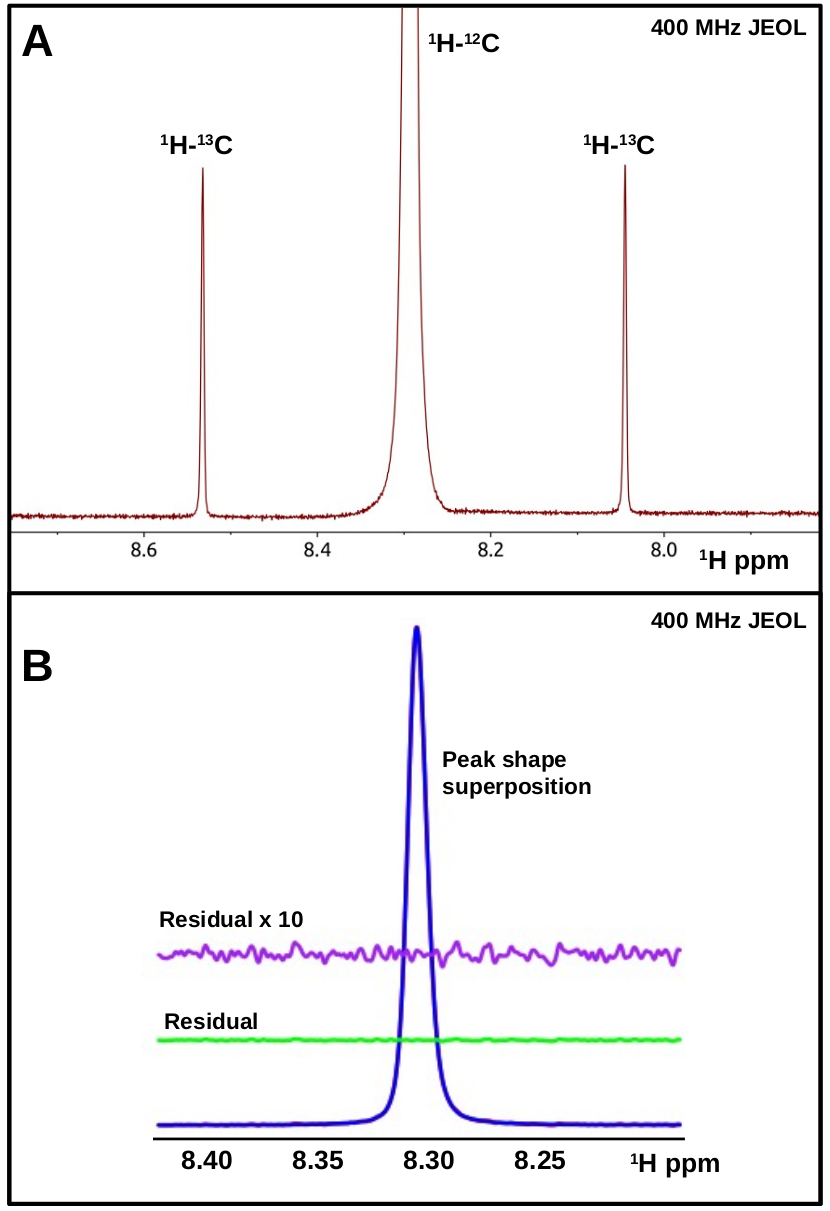


**Figure S4:** Example of an NMR analysis to determine the *R*(¹³C/¹²C) ratio in USGS108 calcium formate using a JEOL 400-MHz spectrometer. Exactly 1536 scans were averaged to produce the spectrum. **(A)** Section of the ¹H NMR spectrum showing the central ¹H-¹²C and satellite ¹H-¹³C peaks. **(B)** The sum of the two ¹H-¹³C satellite peaks (blue) is superimposed on the central ¹H-¹²C peak (red). The *R*(¹³C/¹²C) ratio is the scale factor (*ratio* in eq. 2) for the superposition. The fit is sufficiently close so that the red and blue lines are not separately visible; the residual (difference) between the red and blue lines is shown in green, along with the residual times ten in purple, offset from the baseline for clarity. This NMR analysis using 400 MHz data produced a *R*(¹³C/¹²C) ratio of 0.011258(5) for the USGS108 calcium formate, which is consistent with the 600 MHz data, within the experimental uncertainty. The raw data file is caf_heavy_jeol_01.


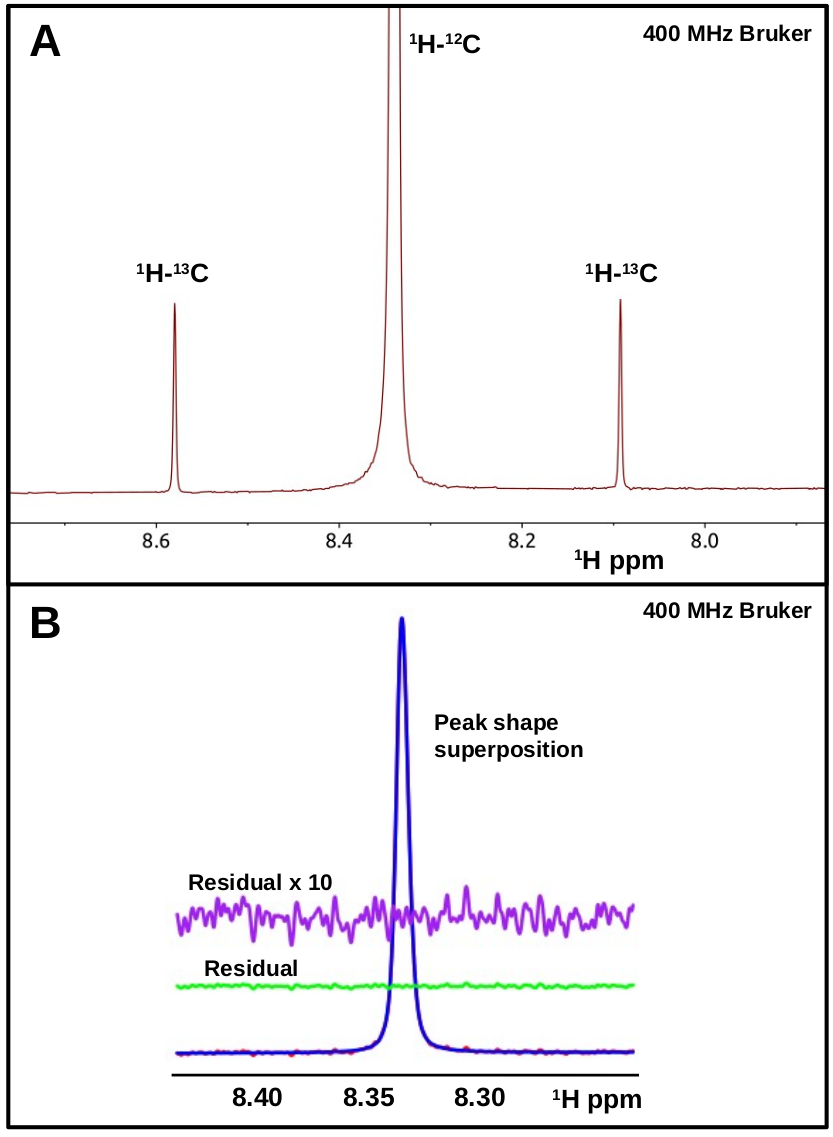


**Figure S5:** Example of an NMR analysis to determine the *R*(¹³C/¹²C) ratio in USGS108 calcium formate using a Bruker 400 MHz spectrometer. Exactly 192 scans were averaged to produce the spectrum. **(A)** Section of the ¹H NMR spectrum showing the central ¹H-¹²C and satellite ¹H-¹³C peaks. **(B)** The sum of the two ¹H-¹³C satellite peaks (blue) is superimposed on the central ¹H-¹²C peak (red). The *R*(¹³C/¹²C) ratio is the scale factor for the superposition. The fit is sufficiently close so that the red and blue lines are not separately visible; the residual (difference) between the red and blue lines is shown in green, along with the residual times ten in purple, offset from the baseline for clarity. This NMR analysis using 400 MHz NMR data produced a *R*(¹³C/¹²C) ratio of 0.011264(10) for USGS108 calcium formate, which is consistent with the 600 MHz data, within the experimental uncertainties. The raw data file is caf_heavy_neo400_02.

Any use of trade, firm, or product names is for descriptive purposes only and does not imply endorsement by the U.S. Government.

**Reference**

1. JCGM 100:2008 Evaluation of measurement data — Guide to the expression of uncertainty in measurement (GUM). Joint Committee for Guides in Metrology (JCGM); 2008.
